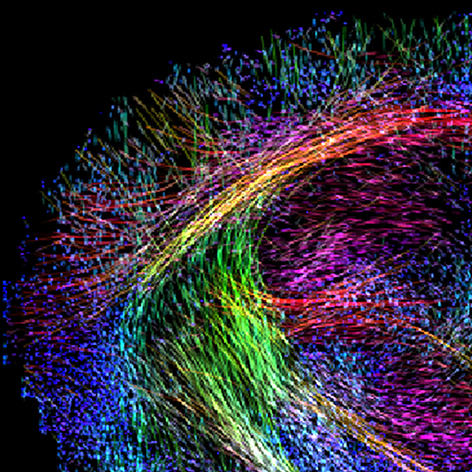# Toxoplasmosis and neuronal changes in mice

**Published:** 2014-04

*Toxoplasma gondii* infects all warm-blooded animals and is associated with specific behavioural and neurological alterations in both rodents and people. To find out how this intracellular parasite alters brain function, Dunay et al. have been investigating the pathological changes in different brain regions of mice chronically infected with *T. gondii*. The researchers report that local connectivity is impaired in these mice, particularly within somatosensory regions. These changes are paralleled by reduced expression of two cytoskeletal proteins in the somatosensory cortex and hippocampus, and reduced dendritic complexity in non-infected neurons from these brain areas in infected mice. Finally, the expression of proteins that regulate key synaptic functions is modified in the same brain regions. These results indicate that *T. gondii* infection induces marked neuroanatomical changes in brain regions that control normal behaviour and establish a murine model for translational studies of chronic toxoplasmosis. Page 459

**Figure:**